# Editorial for Special Issue: Neuroglobin from Brain Protection to Cancer Progression

**DOI:** 10.3390/cells11142181

**Published:** 2022-07-13

**Authors:** Maria Marino, Roberta Misasi, Margherita Ruoppolo

**Affiliations:** 1 Department of Science, University Roma Tre, Viale Guglielmo Marconi 446, 00146 Rome, Italy; 2 Department of Experimental Medicine, University La Sapienza, 00185 Rome, Italy; 3 Department of Molecular Medicine and Medical Biotechnology, School of Medicine, University of Naples Federico II, 80131 Naples, Italy

Since its discovery in 2000 [1] and characterization of its preferential expression in the brain [1,2], neuroglobin (NGB) presented a new opportunity to understand the mechanisms underlying neural pathologies, as well as to discover new therapeutic approaches. Indeed, its active role in brain as an oxidative stress sensor and cytoprotective factor against neurodegeneration, hypoxia, ischemia, toxicity, glutamate toxicity, and nutrient deprivation [2] lead us to consider that NGB is part of endogenous neuroprotective pathways, thus elucidating a new scenario in the possibility of discovering new therapeutic approaches. However, NGB has recently been recognized as a new tumor-associated protein, highlighting the role of this globin in increasing tumor cell resilience against stress-induced apoptotic pathway and the antioxidant systems activated by cancer cells [3]. In fact, these pathways and cellular processes were found unbalanced in NGB-deficient cancer cells [4]. The dual nature of NGB, from protecting the vitality and function of the central nervous system to cancer progression, makes it imperative to better understand the molecular mechanisms in which this globin is involved. In this Special Issue of *Cells* entitled “Neuroglobin from Brain Protection to Cancer Progression,” we collect both reviews and articles aimed to identify the molecular mechanisms at the root of the biological functions of NGB that will bear fundamental and translational significance, especially in the development of therapeutics against neurological disorders.

The brain was believed to lack any system able to promote the storage and/or the diffusion of oxygen similar to what occurs in the muscle system with myoglobin (MB). However, the discovery of NGB as a monomeric intracellular globin has raised the possibility of a myoglobin-like function of NGB [5]. In the paper of De Simone and collaborators [6], the structural and (pseudo-)enzymatic properties of NGB have been reviewed; the researchers attempted, for the first time, to link these properties to each other. Authors showed that NGB actions in vivo depend on its concentration and on an efficient ferric NGB reductase system, still unknown, restoring ferrous NGB. Thus, when present at high concentrations (~100–200 µM), NGB may facilitate O_2_ buffer and transport, whereas at low concentrations (~1 µM) NGB can display only potential enzymatic activities and cell signaling in most tissues and organs. In this review, a role for NGB is highlighted as a compensatory protein responding to hypoxic, ischemic or oxidative injuries by activating survival/antiapoptotic pathways [6]. The pioneering study of Schmidt and collaborators [7] first evidenced that the NGB levels in retinal extracts from mice were 100-fold higher with respect to total brain extracts. They estimated a NGB concentration in the retina in the range of 100 to 200 µM, comparable to MB levels in muscle and accountable for a possible NGB function in the transport or short-term storage of oxygen [6]. The paper of Solar Fernandez and collaborators [8] provides the undercurrent knowledge on NGB distribution in retinal layers and the evidence about the connection between NGB level modulation and the functional outcome in terms of retinal neuroprotection to provide a novel therapeutic/preventive target for visual pathway degenerative disease. Authors reported evidence on the effect of ectopic overexpression of NGB in the retinal tissue, and, in particular, in retinal ganglion cells. High induced levels of NGB have a critical function in preserving retinal neuron loss and vision pathway under different stress conditions/retinal diseases as the main consequence of the globin ability to maintain or enhance mitochondrial functions, supporting the idea of NGB as the main target candidate for preserve or restore retinal tissue during degeneration [8].

Data accumulated to date suggest that NGB is a stress-inducible protein whose overexpression and mitochondrial localization could provide a compensatory response to injuring stimuli protecting against neurodegenerative diseases. Moreover, stably transfected neuroblastoma cells overexpressing endogenous NGB suggested that the role in neuroprotection played by NGB is reliable only through interaction with mitochondrial lipid raft-associated complexes [4,9]. Consequently, treatments that increase NGB levels and/or increase its mitochondrial targeting might have therapeutic value. In the paper of Barreto and colleagues [10], the ability of the hormone 17β-estradiol to upregulate and translocate NGB to the mitochondria to sustain neuronal and glial cell adaptation to injury has been discussed. Notably, the authors reported that the sex-specific regulation of NGB expression in neurons and glial cells has not received enough attention; however, sex differences in the expression of NGB after brain injury have been reported sustaining a possible contribution of the estrogenic regulation of NGB to the generation of sex differences in brain pathology [10].

The paper of Manganelli and colleagues [11] has investigated the involvement of NGB overexpression in increasing energetic metabolism in neuronal derived cells. These authors measured the effect of overexpressed NGB on oxygen consumption rate (OCR), through Seahorse XF technology and on autophagy induction. Proteomic analysis revealed several differentially regulated proteins, involved in oxidative phosphorylation, and integral mitochondrial proteins linked to energy metabolism [4]. Moreover, NGB overexpression increased mitochondrial ATP production enhancing the bioenergetic metabolism, increasing OCR and oxygen consumption [11]. These results highlight the active participation of NGB in several cellular processes that can be upregulated in response to NGB overexpression, playing a role in the adaptive response to stress in neuroblastoma cells.

In their paper, Solar Fernandez and colleagues [12] evaluated the levels and localization of NGB in estrogen receptor positive (ERα+) breast ductal carcinoma tissue of different grades derived from pre-and post-menopausal patients. The reported results indicate a strong association between NGB accumulation, ERα, AKT activation, and the G3 grade, while no association with the menopausal state has been evidenced. Analyses of the data set (e.g., GOBO) strengthen the idea that NGB accumulation could be linked to tumor cell aggressiveness (high grade) and resistance to treatment [12]. These data support the view that NGB accumulation, mainly related to ER expression and tumor grade, represents a compensatory process, which allows cancer cells to survive in an unfavorable environment.

As a whole, this Special issue highlights the dual role played by NGB overexpression in human diseases as well as some of involved mechanisms. Data sustain that NGB has shown enormous potential to develop therapies against inflammation and oxidative stress due to its common pathological features across many neurological diseases, including stroke, Alzheimer’s disease, and traumatic brain injury, among others. On the other hand, NGB overexpression shows similar protective effects on estrogen-related cancer. Perhaps one of the greatest current challenges is to seek strategies that aim to selectively increase both the levels and cellular expression of NGB in the brain, reducing or preventing the globin accumulation in estrogen-related cancer. This strategy could be reached with the use of plant-derived compounds with estrogenic activity, that differently activating estrogen receptor subtypes reduces NGB levels in cancer increasing the globin levels in brain tissues [13]. Investigations of other biological functions this protein may have on human health upon injury ought to be the subject of future studies.
